# Assessing efgartigimod dosing patterns and Myasthenia Gravis Activities of Daily Living outcomes in clinical practice: results from a large patient support program database

**DOI:** 10.3389/fneur.2026.1881758

**Published:** 2026-07-30

**Authors:** Pushpa Narayanaswami, A. Gordon Smith, Ratna Bhavaraju-Sanka, Cynthia Qi, Matthew Jefferson, Jamie Aldridge, Kristin Heerlein, Deborah Gelinas, Mihir Dhingra, Rohit R. Menon, Mai Sato, Gil I. Wolfe

**Affiliations:** 1 Department of Neurology, Beth Israel Deaconess Medical Center/Harvard Medical School, Boston, MA, United States; 2 Department of Neurology, Virginia Commonwealth University, Richmond, VA, United States; 3 Department of Neurology, University of Texas Health Science Center at San Antonio, San Antonio, TX, United States; 4 argenx, Ghent, Belgium; 5 Department of Neurology, UNC School of Medicine, Chapel Hill, NC, United States; 6 ZS Associates, Gurugram, Haryana, India; 7 ZS Associates, Bengaluru, Karnataka, India; 8 ZS Associates, New York, NY, United States; 9 Department of Neurology, Jacobs School of Medicine and Biomedical Sciences, University at Buffalo, Buffalo, NY, United States

**Keywords:** activities of daily living, dosing pattern, efgartigimod, myasthenia gravis, patient support program

## Abstract

**Background:**

Efgartigimod, a neonatal Fc receptor (FcRn) blocker, is approved for the treatment of generalized myasthenia gravis (gMG). Evidence describing large-scale cohorts in routine United States (US) practice remains limited.

**Objective:**

To characterize efgartigimod dosing patterns and Myasthenia Gravis Activities of Daily Living (MG-ADL) outcomes among a US cohort enrolled in the My VYVGART Path patient support program.

**Methods:**

This retrospective cohort study analyzed adults with gMG who initiated efgartigimod on or before December 31, 2025, and had a baseline (pre-efgartigimod) MG-ADL score recorded and ≥4 follow-up assessments available. Outcomes included dosing interval distributions, longitudinal MG-ADL trends, durability of clinically meaningful improvement (CMI; ≥2-point reduction), and best observed response.

**Results:**

Among 3,326 eligible patients (mean [SD] baseline MG-ADL: 8.2 [3.8]; mean [SD] treatment duration: 18.0 [11.0] months), the most common inter-cycle interval length was 4 weeks, though intervals ranged widely, reflecting individualized care. Patients achieved rapid and sustained clinical improvement, with mean MG-ADL improvement of 3.3 points by Month 1 and 4.5 points by Month 18. Of 2,964 patients achieving CMI, the majority (>75%) maintained CMI for >80% of their available follow-up time. Best observed response analyses showed a mean (SD) improvement from baseline of 5.9 (3.5) points, with 83% achieving MG-ADL score ≤4 and 47% achieving minimal symptom expression (MG-ADL score 0–1).

**Conclusion:**

In this large and diverse US cohort, efgartigimod was administered using both proactive (structured) and individualized (flexible) dosing strategies, leading to rapid, deep, and sustained clinically meaningful improvements. These findings support efgartigimod as an effective and adaptable therapy for gMG in routine clinical practice.

## 1 Introduction

Generalized myasthenia gravis (gMG) is a rare autoimmune disorder characterized by fluctuating, fatigable muscle weakness caused by autoantibodies targeting the neuromuscular junction. The most common target is the acetylcholine receptor (AChR), accounting for ~85% of cases, with less frequent involvement of muscle-specific kinase (MuSK) or low-density lipoprotein receptor-related protein 4 (LRP4) (1). gMG is a highly heterogeneous disease in its presentation and disease course, with a broad spectrum of symptoms including diplopia, ptosis, varying degrees of muscle weakness of the trunk and limbs, and bulbar and respiratory muscle involvement (2). The management of gMG aims for durable symptom control through a diverse array of therapeutic options (3, 4). Conventional treatments include symptomatic agents like acetylcholinesterase inhibitors (AChEis), along with immunomodulatory therapies such as glucocorticoids (GCs), nonsteroidal immunosuppressants (NSISTs), and in some cases, immunoglobulins or plasma exchange (3, 4).

The significant side-effect burden and incomplete efficacy of many conventional agents, particularly long-term GCs, have driven a paradigm shift in gMG care (5–8). The recent approval of targeted therapies has fundamentally expanded the treatment landscape. These mechanism-directed options include neonatal Fc receptor (FcRn) blockade (efgartigimod, rozanolixizumab, nipocalimab), complement C5 inhibition (eculizumab, ravulizumab, zilucoplan), and B cell depletion (inebilizumab) (5, 7). This diversification offers the potential for earlier and perhaps more effective disease control with potentially improved safety profiles, expanding opportunities to individualize patient care.

Efgartigimod, an engineered Fc fragment with high affinity for FcRn, reduces pathogenic IgG levels by competitively blocking FcRn-mediated IgG recycling, thereby enhancing lysosomal degradation of unbound IgG (9). It is approved for use in gMG in the United States (US), European Union (EU), Japan, Canada, China, Australia, Switzerland, and the United Kingdom (UK), with regional differences in antibody-status requirements. Pivotal evidence from the ADAPT and ADAPT-SC trials established the safety and efficacy of both intravenous and subcutaneous efgartigimod formulations in gMG using flexible dosing strategies (10, 11). While these trials have been foundational, large-scale evidence on the long-term application of this therapy in routine clinical practice is still emerging. The transition from a structured trial environment to the heterogeneity of clinical management creates a need to understand actual dosing patterns and the associated outcomes in this setting. While a growing body of global evidence in practice has provided valuable early insights into the use of efgartigimod (12–19), a large-scale, comprehensive analysis detailing dosing patterns, as well as depth and durability of response, across a diverse, nationally representative US cohort is not available. The My VYVGART Path patient support program (PSP) provides a unique opportunity to address this evidence gap. This study aimed to characterize 3 key aspects of efgartigimod use in a large, diverse US patient population: efgartigimod dosing patterns, including cycle structure and inter-cycle interval length; clinical outcomes, including both overall improvement and the depth and durability of response measured through Myasthenia Gravis Activities of Daily Living (MG-ADL); and potential associations between certain patient characteristics and MG-ADL outcomes.

## 2 Materials and methods

### 2.1 Study design and data source

This retrospective observational cohort study evaluated adults with gMG who initiated efgartigimod in routine clinical practice in the US. Clinical and dosing data were obtained from the My VYVGART Path PSP, an argenx-sponsored program designed to assist patients with treatment access (e.g., benefit verification, insurance requirements, infusion center coordination) and to support ongoing therapy through nurse case manager (NCM) outreach. Nearly 9,000 patients with gMG were enrolled at the time of this study, with over 30,000 MG-ADL assessments captured, enabling large-scale assessment of treatment patterns and outcomes in routine US clinical practice.

NCMs collected patient-reported information, including demographics, efgartigimod infusion schedules, and MG-ADL scores, through periodic structured phone contact subject to patient preferences and availability. All data included in this analysis were de-identified before extraction from the PSP database. This secondary analysis received exemption from the Institutional Review Board (Advarra, Inc., Columbia, MD, USA) and was conducted in accordance with the local legislation and institutional requirements.

### 2.2 Study population and selection bias assessment

Efgartigimod infusion and dosing data were reliably recorded as part of treatment coordination activities in the PSP. However, because the PSP’s primary purpose is patient assistance rather than research, MG-ADL scores were obtained only when patients were reachable and willing to provide updates, resulting in inherently varied capture from patient to patient.

Given these considerations, eligible patients for this study were adults (≥18 years) with gMG who initiated efgartigimod on or before December 31, 2025, and fulfilled both of the following criteria: (1) a recorded baseline MG-ADL score prior to the first infusion, and (2) at least 4 MG-ADL assessments captured in the PSP data at any point after efgartigimod initiation. The requirement for ≥4 post-baseline MG-ADL scores was essential to ensure that each patient contributed sufficient longitudinal information despite variable assessment captures.

To characterize possible selection bias introduced by this requirement, baseline characteristics of the analytic cohort (*N* = 3,326) were compared with those of the remaining 5,595 PSP enrollees excluded due to insufficient number of follow-up MG-ADL assessments (Supplementary Table 1). This comparison supports transparent acknowledgment that the analytic cohort represents a specific subpopulation of patients (expected to have persistent program participation and clinical follow-up) by virtue of requiring sufficient outcome data availability for analysis.

Additionally, due to the nature of the PSP dataset, potential responder enrichment can be expected; patients followed up for a longer time may be more likely to show favorable long-term treatment responses and higher treatment adherence. To address whether the cohort composition changed systematically over time, key parameters were compared between patients who were followed up for varied lengths (Supplementary Table 2).

### 2.3 Dosing patterns

Efgartigimod is dosed as 4 once-weekly infusions, constituting a ~3-week treatment period. Subsequent treatment periods occur after individualized intervals determined by clinical evaluation (20, 21). In this study, (1) a treatment “period” was defined as the time from first to final infusion during the 4 once-weekly dosing; (2) a treatment “interval” was defined as the time between the final infusion of 1 treatment period and the first infusion of the next; and (3) a treatment “cycle” was defined as 1 treatment period plus the subsequent interval (e.g., a 4-week interval corresponds to a 7-week cycle). All measurable intervals flanked by 2 treatment periods (including those associated with patients who later discontinued or paused therapy) were included to provide a comprehensive view of dosing variability in routine clinical practice. For all dosing and outcomes analyses, patients were pooled regardless of efgartigimod formulation used (intravenous, subcutaneous, or both).

### 2.4 Clinical outcomes

**Figure 1:**
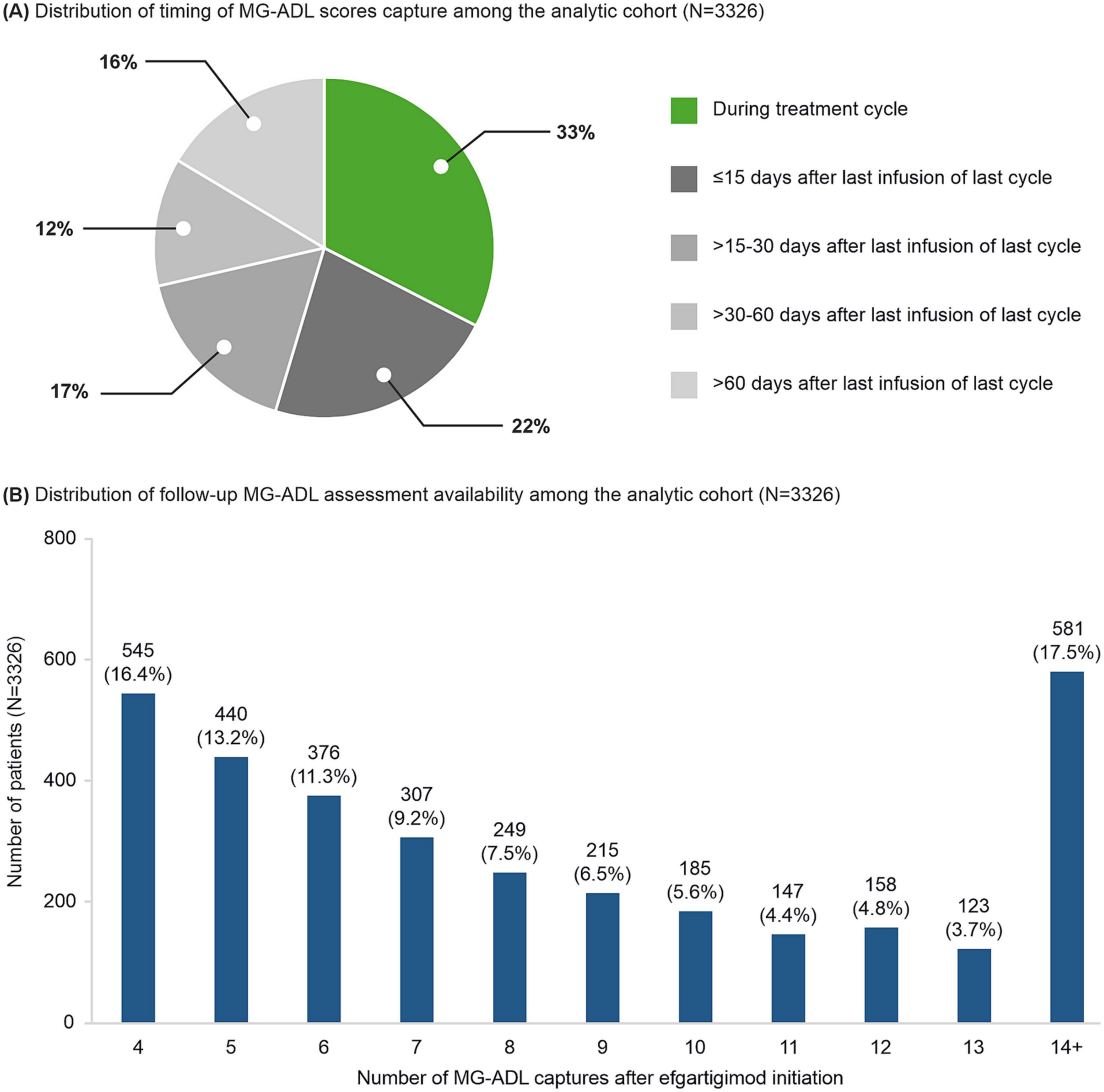
Distribution of MG-ADL assessments in PSP data. **(A)** Distribution of timing of MG-ADL score capture among the analytic cohort (*N* = 3,326). **(B)** Distribution of follow-up MG-ADL assessment availability among the analytic cohort (*N* = 3,326).

MG-ADL is an 8-item patient-reported outcome scale (range: 0–24; higher scores indicate greater symptom severity) widely used in gMG clinical trials and observational studies to measure clinical efficacy (22). A key challenge in assessing gMG is the inherent variability of its symptoms, which can fluctuate significantly day to day. To control for this, clinical trials typically measure MG-ADL scores at prespecified, uniform timepoints. In contrast, data in this PSP cohort were collected asynchronously, reflecting actual patient–nurse interactions rather than a rigid study schedule. As a result, MG-ADL assessments were captured at heterogeneous timepoints relative to treatment cycles (Figure 1A).

This fundamental difference in data structure meant that standard analyses based on fixed timepoints could not be solely relied upon. Therefore, we adopted a multifaceted analytical approach to characterize the treatment journey, assessing outcomes from several complementary perspectives:

#### 2.4.1 Longitudinal trend

Population-level symptom trends were examined by summarizing the mean MG-ADL improvement from baseline at monthly intervals (up to 18 months after efgartigimod initiation) and by treatment cycles (up to 12 cycles). If multiple MG-ADL values were recorded within a given month or cycle, only the lowest (best) value was used within that interval. As the average cadence of MG-ADL assessments recorded in the PSP was once every ~2 months while on treatment, patients having more than 1 MG-ADL assessment available in any given time window was rare. A sensitivity analysis was performed using the mean value of all available MG-ADL scores within each window (month or cycle), taking the difference between each captured MG-ADL score and the patient’s baseline, and averaging the improvements across all assessments within the window.

#### 2.4.2 Long-term durability

A clinically meaningful improvement (CMI) was defined as a ≥2-point reduction from baseline MG-ADL score. For each patient achieving CMI at least once after efgartigimod initiation, the proportion of time spent in CMI was calculated as:


$$\frac{\mathrm{Sum}\;\text{of}\;\mathrm{all}\;\text{time periods}\;(\text{in days})\;\text{where patient was in}\;a\;\mathrm{CMI}\;\text{state}}{\begin{array}{c} \text{Total days between the date of first recorded}\;\mathrm{CMI}\;\text{to the date of}\;\\ \text{last available}\;\mathrm{MG}-\mathrm{ADL}\;\text{assessment} \end{array}}$$

Each recorded MG-ADL score was assumed to persist until the next observation.

#### 2.4.3 Best observed response

To apply a consistent framework to measure the maximal clinical benefit achieved by each patient, independent of the variable timing and frequency of their MG-ADL assessments, we analyzed each patient’s best observed response as an exploratory outcome. This was defined as the lowest MG-ADL score observed for each patient at any timepoint during their follow-up. While we acknowledge this metric is susceptible to influence by assessment frequency and does not measure durability, its strength lies in applying a uniform analytical framework to every patient in this large, heterogeneous cohort, allowing for a standardized assessment of the treatment’s capacity to induce a deep clinical response.

Best observed response metrics included the largest observed MG-ADL improvement from baseline (total and by subdomain), and the best disease state achieved (including the proportion attaining MG-ADL score ≤4 or minimal symptom expression [MSE], defined as MG-ADL score of 0 or 1) at any time after efgartigimod initiation.

### 2.5 Exploratory subgroup analyses

Prespecified exploratory subgroup analyses were conducted to evaluate the consistency of treatment response. These analyses remained descriptive in nature, intended to uncover potential directional patterns and associations, as key clinical information required for a rigorous multivariate model (such as concomitant gMG treatment usage) to assess independent drivers was missing in the dataset. Results and statistical analyses were not adjusted and purely limited to descriptive statistics.

Due to the heterogeneity of MG-ADL assessment timing in this dataset, outcomes were evaluated from 2 perspectives. Durability was primarily assessed using the mean proportion of each patient’s follow-up time spent in CMI. To provide a consistent measure of response magnitude across all patients, “best observed response” metrics (achievement of MSE at any time and mean largest MG-ADL improvement) were also evaluated. Subgroups were stratified based on 2 categories: (1) fixed baseline or clinical characteristics (e.g., age, sex, prior gMG treatments, and baseline MG-ADL score) and (2) on-treatment characteristics that evolved over time (e.g., average interval length, number of cycles, and treatment duration). Patients who reported prior exposure to intravenous or subcutaneous immunoglobulin (IVIG/SCIG), plasma exchange (PLEX), C5 inhibitors, FcRn blockers, and/or rituximab were grouped as having had exposure to “advanced therapy.”

### 2.6 Statistical analysis

Descriptive statistics were used to summarize patient characteristics, dosing patterns, and MG-ADL outcomes. Means, standard deviations, and medians were calculated for continuous variables; counts and percentages were used for categorical variables. Differences across subgroups were assessed using Student’s t-test or one-way ANOVA for continuous outcomes and Chi-square tests for categorical comparisons. Multiplicity adjustments were not applied, as subgroup analyses were exploratory in nature (descriptive associations). Two-sided *p*-values <0.05 were considered statistically significant. All statistical analyses were performed using Python version 3.10.6.

## 3 Results

### 3.1 Patient selection and characteristics

The study included 3,326 patients with gMG in the US who initiated efgartigimod, whose baseline characteristics reflected the clinical and healthcare system heterogeneity observed in routine MG care (Table 1). The cohort spanned a broad spectrum of disease severity, a key indicator of clinical diversity: baseline MG-ADL score was median (IQR [interquartile range]) 8 (5.3–11.0), with 65% (*n* = 2,171) of patients presenting with moderate-to-severe disease (baseline MG-ADL score ≥7). Based on patient self-reporting, 81% (*n* = 2,700) had received at least 1 prior gMG treatment.

**Table 1:** Baseline characteristics of analytic cohort.

Overall	*N* = 3,326
Age	
Mean (SD), years	65.7 (14.9)
Median (IQR), years	69 (58–77)
Range, years	18–89
Distribution, n (%)	
18 to <50 years	493 (14.8)
50 to <65 years	738 (22.2)
≥65 years	2,095 (63.0)
Sex	
Female, n (%)	1,323 (39.8)
Weight^a^	
Mean (SD), kg	93.7 (28.4)
Median (IQR), kg (range)	90.1 (75.0–108.0) (27–280)
Prescriber US region, n (%)	
South	1,477 (44.4)
Midwest	538 (16.2)
West	646 (19.4)
Northeast	529 (15.9)
Not available	136 (4.1)
Insurance, n (%)^b^	
Commercial	1,042 (31.3)
Government	2,393 (71.9)
Other	122 (3.7)
Prior gMG treatments, n (%)^c^	
AChEi	1,454 (43.7)
Any NSIST	680 (20.4)
Any GC	1765 (53.1)
Any advanced therapy^d^	1,668 (50.2)
Efgartigimod formulation used at any time, n (%)^e^	
IV only	2,106 (63.3)
SC only	559 (16.8)
Both	661 (19.9)
Baseline MG-ADL score	
Mean (SD)	8.2 (3.8)
Median (IQR) (range)	8 (5.3–110) (0–22)
Distribution, n (%)	
0–4 points	563 (16.9)
5–9 points	1,584 (47.6)
10–24 points	1,179 (35.4)

a Weight information was available for only 2,542/3,326 patients.

b Categories may not add up to the total as patients may have been tagged to multiple payers.

c Prior treatment information was available for only 2,700/3,326 patients and was self-reported. Proportions may not add up to 100% as patients could have had multiple concomitant prior therapies. Additionally, some patients may not have listed all their prior treatments, e.g., only their most recent.

d Advanced therapies included IVIG/SCIG, PLEX, C5 inhibitors, FcRn blockers, and rituximab.

e Both VYVGART Hytrulo formulations, i.e., SC and PFS, considered under SC formulation.

AChEi, acetylcholinesterase inhibitor; FcRn, neonatal Fc receptor; GC, glucocorticoid; gMG, generalized myasthenia gravis; IQR, interquartile range; IV, intravenous; IVIG, intravenous immunoglobulin; MG-ADL, Myasthenia Gravis Activities of Daily Living; NSIST, nonsteroidal immunosuppressive therapy; PFS, pre-filled syringe; PLEX, plasma exchange; SC, subcutaneous; SCIG, subcutaneous immunoglobulin; SD, standard deviation; US, United States. The most recent score captured on or before EFG initiation is considered to be the baseline score.

The diversity of the cohort extended beyond clinical factors (Table 1). All US regions were represented, with data sourced from >1800 unique prescribers across all 50 US states, as well as the District of Columbia and Puerto Rico. Furthermore, the population included a mix of insurance types, with 31% (*n* = 1,042) covered by commercial plans, 72% (*n* = 2,393) by government plans including Medicare and Medicaid, and 4% (*n* = 122) by other plans. The mean (SD) age was 65.7 (14.9) years, with 15% of patients below 50 years, 22% between 50 and 65 years, and 63% ≥65 years of age; 40% of the cohort was female.

Compared with the remaining efgartigimod initiators enrolled in the PSP who were excluded, the analytic cohort generally reflected patients who initiated efgartigimod sooner after its approval. As detailed in Supplementary Table 1, these groups were broadly similar in age, sex distribution, prior gMG treatment experience, and baseline MG-ADL severity. However, the analytic cohort had different regional distribution with slightly more representation of government insurance and, importantly, was more likely to have initiated efgartigimod sooner after approval, compared to the excluded cohort, reflecting enrichment for patients with longer treatment duration and follow-up. Aside from enriching for patients who initiated efgartigimod before 2024–2025, longer time on treatment did not have a major impact on basic demographics, baseline disease severity, and efgartigimod response metrics, suggesting the cohort composition was relatively consistent over time (Supplementary Table 2).

### 3.2 Dosing patterns

**Figure 2:**
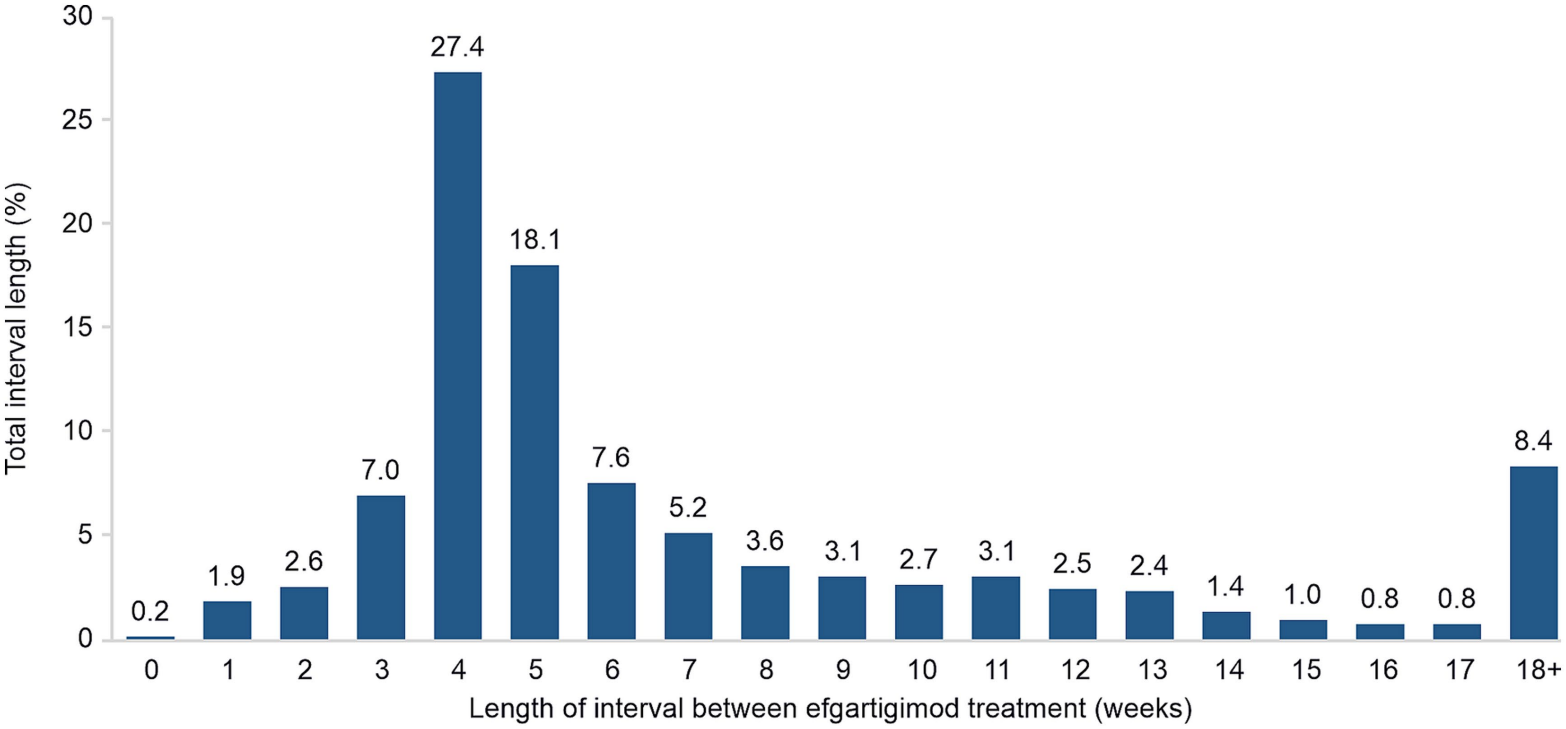
Distribution of interval lengths during efgartigimod treatment.

For the overall analytic cohort, efgartigimod treatment duration was median (IQR) 15.7 (8.8–25.6) months (Table 2). Among the 54% (*n* = 1800) who had been treated with efgartigimod for at least 1 year with complete dosing information, the observed median (IQR) annualized cycles completed during the first year of treatment was 5 (4–6.5) cycles. A majority of the total cohort (96%; *n* = 3,187) had completed at least Cycle 1 and were eligible for the dosing pattern analysis. Among them, the length of treatment intervals was median (IQR) 5 (4–9) weeks (Table 2). Four weeks was the most common interval length (27%), followed by 5 weeks (18%), translating into 7 or 8 weeks as the most common cycle lengths. 12% of the intervals were shorter than 4 weeks, while 43% of the intervals were 6 weeks or longer (Figure 2).

**Table 2:** Efgartigimod dosing characteristics.

Overall	*N* = 3,326
Efgartigimod treatment duration^a^	
Mean (SD), months	18.0 (11.0)
Median (IQR), months	15.7 (8.8–25.6)
Range, months	0.7–46.5
Distribution, n (%)	
<3 months	89 (2.7)
3- <6 months	365 (11.0)
6- <12 months	811 (24.4)
12- <18 months	606 (18.2)
18+ months	1,455 (43.7)
Time of efgartigimod initiation, n (%)	
2022	686 (20.6)
2023	1,228 (36.9)
2024	917 (27.6)
2025	495 (14.9)
Number of maximum efgartigimod treatment cycles completed, n (%)	
1	331 (10.0)
2	429 (12.9)
3	376 (11.3)
4	342 (10.3)
≥5	1,709 (51.4)
Treatment interval lengths^b^	
Mean (SD), weeks	8 (8)
Median (IQR), weeks	5 (4–9)
Range, weeks	0–126
Top 3 most common intervals, n (%)	
4 weeks	5,261 (27.4)
5 weeks	3,475 (18.1)
6 weeks	1,457 (7.6)

a Difference between efgartigimod initiation date and the latest efgartigimod infusion date or MG-ADL capture date, whichever was recorded the most recently.

b Percentages were calculated against the total of 19,224 distinct measurable intervals between treatment cycles identified among the 3,187/3,326 (96%) patients who had completed at least Cycle 1.

IQR, interquartile range; MG-ADL, Myasthenia Gravis Activities of Daily Living; SD, standard deviation.

### 3.3 Clinical outcomes

#### 3.3.1 Longitudinal trend

**Figure 3:**
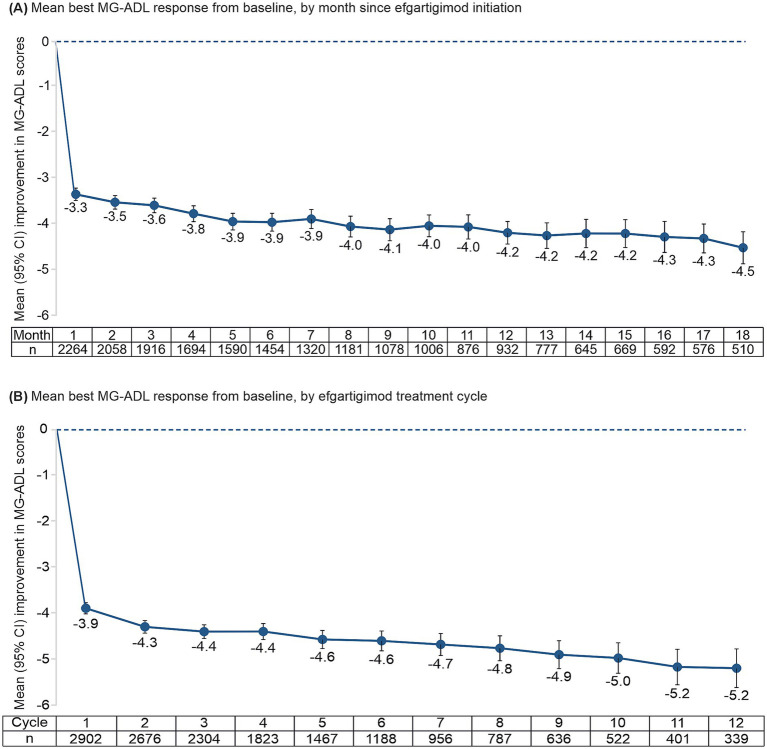
MG-ADL response by month and by cycle. **(A)** Mean best MG-ADL response from baseline, by month since efgartigimod initiation. **(B)** Mean best MG-ADL response from baseline, by efgartigimod treatment cycle.

A total of 32,179 MG-ADL assessments were available after efgartigimod initiation in the analytic cohort, with an average of 10 (range: 4–104) records per patient (Figure 1B). At the population level, a rapid response was observed, with a mean MG-ADL score improvement of 3.3 points from baseline achieved within the first month. This improvement was not only sustained but gradually deepened over the follow-up period, reaching a mean improvement of 4.5 points from baseline by Month 18 (Figure 3A). The trend was similar when analyzed by treatment cycle, with the mean MG-ADL score improvement from baseline increasing from 3.9 points in Cycle 1 to 5.2 points by Cycle 12 (Figure 3B). A similar trend was observed in the sensitivity analysis, where all available MG-ADL scores were included (Supplementary Figure 1). Additionally, a similar trend was observed among a sub-cohort of 1,455 patients (44%) with at least 18 months of efgartigimod treatment experience (Supplementary Figure 2).

#### 3.3.2 Long-term durability

To assess the durability of treatment response, we analyzed 2,964 patients who achieved CMI at least once. This cohort was followed for a median (IQR) 12.2 (6.9–22.7) months after their initial CMI achievement, to evaluate the persistence of benefit (Table 3). MG-ADL scores were captured on average every ~56 days at non-specific timepoints, based on patient willingness and availability (Supplementary Table 3). As CMI state was assumed to be maintained between score captures in this analysis, the proportion of time spent in CMI could be overestimated.

**Table 3:** Proportion of time in CMI (*n* = 2,964).

Patients who achieved CMI at least once, with MG-ADL available after achieving CMI, n (%)^a^		*n* = 2,964 (98.6)
Cohort-level statistics		
Time from efgartigimod initiation to first CMI, months	Mean (SD)	2.2 (3.4)
	Median (IQR)	1 (0.5–2.4)
Available follow-up time after first CMI, months	Mean (SD)	15.3 (10.2)
	Median (IQR)	12.2 (6.9–22.7)
Time maintained in CMI after first CMI, months	Mean (SD)	13.2 (9.9)
	Median (IQR)	10.6 (5.1–19.5)
Proportion of follow-up time after first CMI maintained in CMI, %	Mean (SD)	85.6 (24.1)
	Median (IQR)	100 (80.5–100.0)
Patient-level distribution, n (%)		
Proportion of patients with x% of their time after first CMI maintained in CMI		
100%		1,560 (52.6)
≥90%		1996 (67.3)
≥75%		2,311 (78.0)

2,964 patients out of the analytic cohort of N = 3,326, were eligible for this analysis. Of the analytic cohort, 3,006 (90.4%) patients achieved CMI at least once, and 2,964 (89.1%) had at least 1 MG-ADL score recorded after their first CMI achievement. 42 patients were excluded as they did not have any additional MG-ADL scores recorded after their first CMI.

CMI, clinically meaningful improvement; IQR, interquartile range; MG-ADL, Myasthenia Gravis Activities of Daily Living; SD, standard deviation.

**Figure 4:**
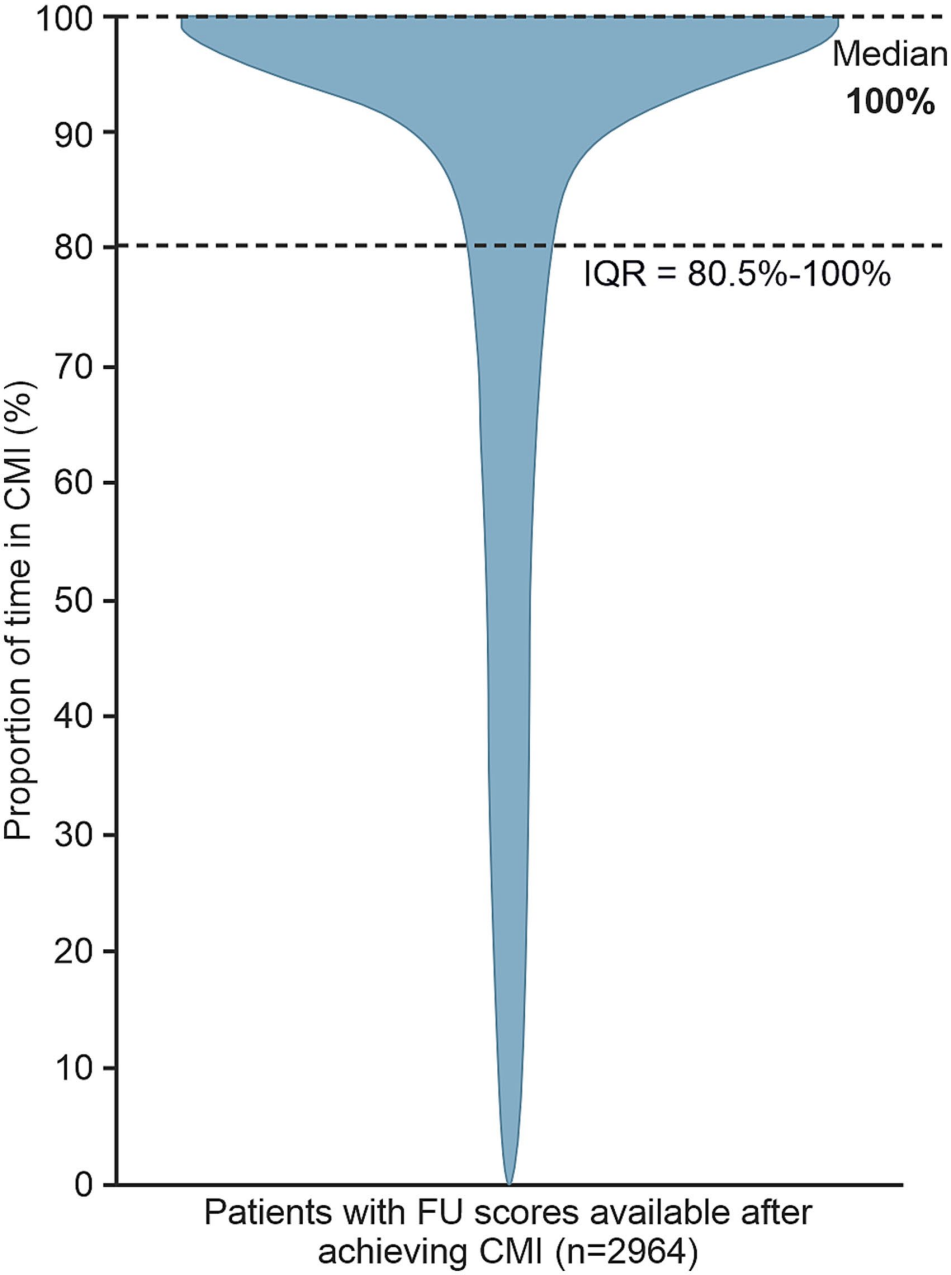
Violin plot distribution of proportion of follow-up time spent in CMI (*n* = 2,964).

Within this follow-up period, robust durability of response was observed, with the median (IQR) proportion of time spent in CMI being 100% (80.5–100.0%; Figure 4). This reflects the finding that more than half of patients (52.6%) remained in a state of CMI for their entire subsequent follow-up period. Furthermore, a majority (78%) of patients spent more than 75% of their follow-up time maintaining CMI (Figure 4; Table 3). Results were similar when calculated based on each MG-ADL assessment after first CMI (Supplementary Table 3). This durable state was also achieved rapidly, with patients reaching their first CMI at a median (IQR) of just 1.0 (0.5–2.4) month after initiating therapy.

#### 3.3.3 Best observed response

**Figure 5:**
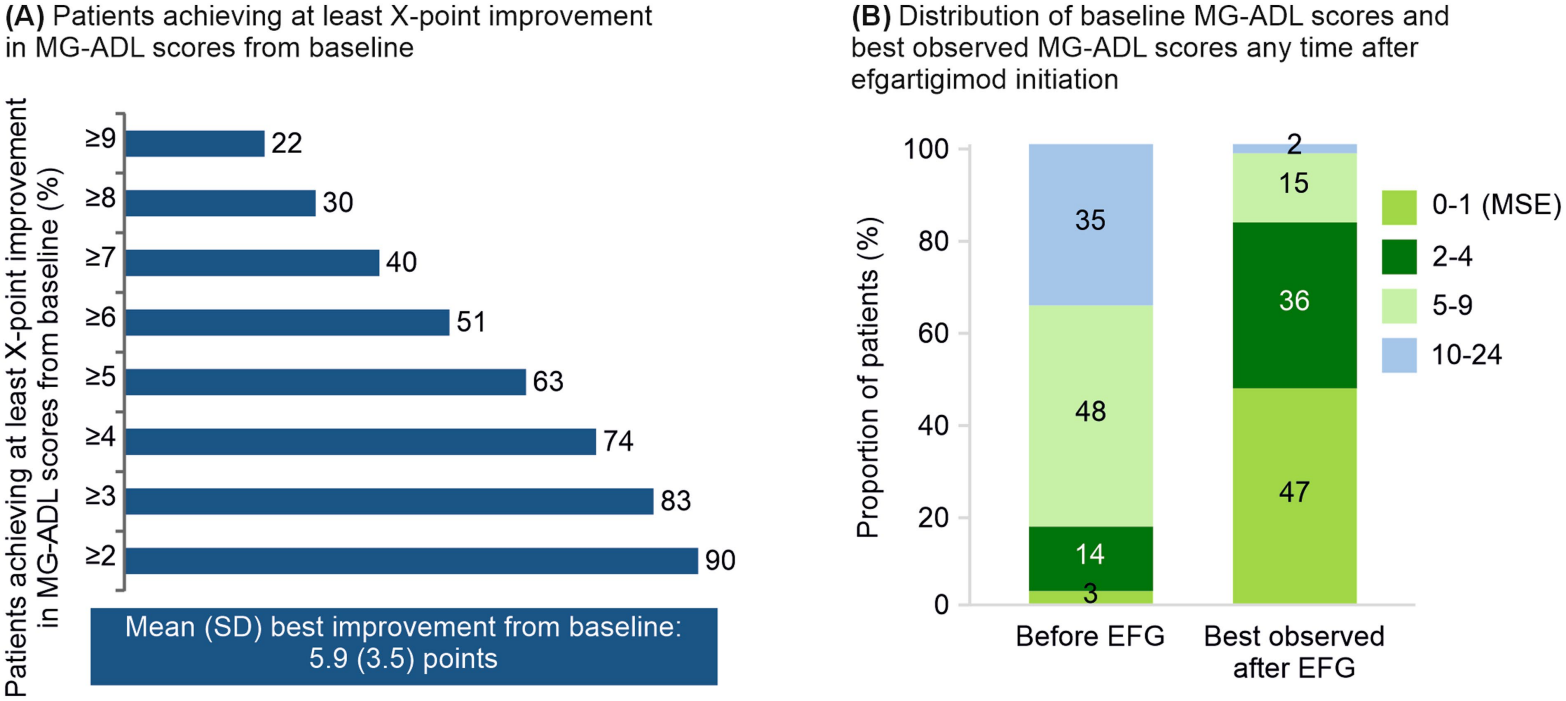
Best observed response. **(A)** Proportion of patients by minimum point improvement in MG-ADL scores from baseline. **(B)** Distribution of baseline MG-ADL scores and best observed MG-ADL scores any time after efgartigimod initiation.

Each patient’s lowest MG-ADL score after efgartigimod initiation was analyzed. A shift toward lower disease severity was observed after efgartigimod initiation, with the largest MG-ADL improvement being mean (SD) 5.9 (3.5) points from baseline (Figure 5A). At least a 60% reduction from baseline was observed across all MG-ADL subdomains, with greatest improvements in bulbar, followed by ocular, limb/gross motor, and respiratory subdomains (Supplementary Table 4). At the point of maximal improvement, 83% of patients achieved an MG-ADL score of 4 or lower, and 47% achieved MSE at least once during their follow-up (Figure 5B).

### 3.4 Exploratory subgroup analyses

Unadjusted, descriptive subgroup analyses suggested that the nature of the treatment response was most strongly associated with baseline disease severity (baseline MG-ADL), which was a key determinant of the type of clinical benefit observed (Table 4; Supplementary Table 5). Regarding best observed response, an inverse relationship was observed where patients with lower baseline MG-ADL (0–4) were most likely to achieve MSE (75.1%), whereas patients with higher baseline MG-ADL (10–24) experienced the largest magnitude of MG-ADL improvement. Regarding response durability, patients with higher baseline MG-ADL spent a larger proportion of follow-up time maintaining CMI versus those with lower baseline MG-ADL scores (likely due to a floor effect).

**Table 4:** Subgroup analysis.

Patient characteristics	n	Mean baseline MG-ADL score	Mean largest observed MG-ADL response from baseline	*p*	Mean (SD) proportion of follow-up time spent in CMI, days (*n* = 2,964)	*p*	Proportion achieving MSE, n (%)	*p*
Overall	3,326	8.2	−5.9		85.6 (24.1)		1,571 (47.2)	
Age, years								
18 to <50	493	8.7	−6.4	<0.001	84.2 (24.6)	0.42	247 (50.1)	0.36
50 to <65	738	8.9	−6.5		85.8 (23.7)		341 (46.2)	
≥65	2095	7.8	−5.5		85.9 (24.0)		983 (46.9)	
Sex								
Male	1744	7.8	−5.7	0.003	86.0 (24.2)	0.24	877 (50.3)	<0.001
Female	1,323	8.7	−6.1		85.6 (23.8)		566 (42.8)	
Unknown	259	8.3	−5.8		83.1 (24.6)		128 (49.4)	
Prior gMG treatment^a^								
Any advanced therapy	1,668	8.6	−6.1	0.24	85.2 (24.2)	0.99	742 (44.5)	0.16
Any other gMG treatments captured	2,289	8.2	−5.9		85.3 (24.3)		1,079 (47.1)	
Not known/thymectomy	857	8.6	−6.1		85.2 (24.4)		379 (44.2)	
Baseline MG-ADL score								
0–4	563	2.7	−1.9	<0.001	69.8 (31.7)	<0.001	423 (75.1)	<0.001
5–9	1,584	7.1	−5.2		84.3 (24.5)		796 (50.3)	
10–22	1,179	12.2	−8.8		91.7 (18.0)		352 (29.9)	
Average interval length^b^								
<4 weeks	142	8.1	−5.9	0.46	84.6 (24.0)	0.76	72 (50.7)	0.01
4–6 weeks	947	8.3	−5.8		85.5 (24.3)		411 (43.4)	
≥6 weeks	2098	8.2	−6.0		86.0 (23.5)		1,032 (49.2)	
Number of efgartigimod cycles completed								
1	331	8.1	−5.0	0.11	86.4 (25.6)	0.53	132 (39.9)	<0.001
2	429	8.2	−5.4		84.3 (24.9)		162 (37.8)	
3	376	8.1	−5.6		85.5 (24.1)		166 (44.1)	
4	342	7.7	−5.5		87.4 (22.8)		153 (44.7)	
≥5	1709	8.3	−6.4		85.8 (23.2)		902 (52.8)	
Efgartigimod treatment duration^c^								
<3 months	89	8.4	−4.6	0.12	84.5 (28.2)	0.51	23 (25.8)	<0.001
3- < 6 months	365	8.2	−5.0		83.8 (26.9)		120 (32.9)	
6- < 12 months	811	8.0	−5.4		86.7 (23.5)		346 (42.7)	
12- < 18 months	606	8.1	−5.7		85.6 (24.3)		269 (44.4)	
18 + months	1,455	8.3	−6.5		85.5 (23.4)		813 (55.9)	

*p*-values were calculated using independent t-test for continuous variables and Chi-square test for categorical variables. *p*-values of <0.05 were considered statistically significant. Multiplicity adjustments were not applied, as subgroup analyses were exploratory in nature (descriptive associations). AChEi, acetylcholinesterase inhibitor; CMI, clinically meaningful improvement; FcRn, neonatal Fc receptor; gMG, generalized myasthenia gravis; IVIG, intravenous immunoglobulin; MG-ADL, Myasthenia Gravis Activities of Daily Living; MSE, minimal symptom expression; NSIST, nonsteroidal immunosuppressive therapy; PLEX, plasma exchange; SCIG, subcutaneous immunoglobulin.

a Prior gMG treatment information was available for only 2700/3326 patients and was self-reported. Advanced therapies included IVIG/SCIG, PLEX, C5 inhibitors, FcRn blockers, and rituximab. Patients who reported any prior treatments that were not advanced therapies (any AChEi, any steroids, and/or any NSISTs) were grouped into “Any other gMG treatments captured” for this analysis.

b Average of all cycle intervals at patient level.

c Difference between efgartigimod initiation date and the latest efgartigimod infusion date or MG-ADL capture date, whichever was recorded the most recently.

The most recent score captured on or before EFG initiation is considered to be the baseline score.

Patients with a longer treatment duration (≥18 months) or a higher number of completed cycles (≥5) had a significantly higher likelihood of achieving MSE versus those with shorter treatment experience (55.9 and 52.8%, respectively; *p* < 0.001). Significant differences (*p* < 0.05) were also observed by age and sex, which may be confounded by the strong impact of baseline disease severity observed. No significant trends were observed for prior gMG treatment.

## 4 Discussion

### 4.1 Summary of key findings

This analysis of over 3,300 US patients from the My VYVGART Path PSP provides a large and comprehensive assessment of efgartigimod dosing and effectiveness in clinical practice. This study sought to evaluate how efgartigimod is used outside of restrictive trial protocols, in a broad population with diverse clinical needs. Our findings demonstrate that in routine clinical practice, efgartigimod was most commonly administered using approximately 4-week intervals, with rapid, substantial, and clinically meaningful improvements sustained over the majority of available follow-up.

### 4.2 Dosing patterns: a shift toward proactive and individualized care

Efgartigimod dosing pattern data from clinical practice offer an important complement to the structured design of the pivotal ADAPT and ADAPT-SC trials (10, 11). While ADAPT was intentionally designed to allow symptoms to return between treatment cycles, a key goal in practice is to maintain continuous disease control. Our data reflects this objective: patients demonstrated sustained clinical benefit over time without the cyclical troughs observed in the trial setting, suggesting that clinicians are successfully tailoring treatment to individual needs. The most common interval between cycles was 4 weeks, suggesting a widespread shift toward simplified, scheduled regimens such as the 4–3-3 paradigm (i.e., a fixed regimen of 4 weekly infusions [over 3 weeks] followed by a 4-week gap [translating to 7-week cycles] for the first 3 cycles) described by Silvestri and colleagues (23) and ADAPT NXT (24). The prevalence of 4-week intervals in our dataset suggests that many clinicians may be independently adopting this intuitive, proactive approach. Logistical factors such as infusion scheduling and healthcare system constraints likely reinforce such use of fixed intervals, creating alignment between clinical strategy and operational feasibility.

Importantly, this trend toward a more structured regimen may be balanced with considerable flexibility. We observed that 12% of intervals were shorter than 4 weeks, and 43% of intervals lasted 6 weeks or longer (median [IQR] interval 5 [4–9] weeks). As the PSP dataset was not able to distinguish clinician-driven optimization of dosing interval lengths from scheduling limitations, access and payer-related factors, treatment pauses, or incomplete data capture, overinterpretation should be cautioned. Nevertheless, these patterns may at least in part suggest clinicians are actively individualizing dosing strategies based on patient need, as demonstrated in other reports (12). This dual approach highlights a sophisticated strategy in clinical settings: adopting predictable, structured regimens to maintain stability for many, while leveraging flexibility to tailor dosing for others. Of note, intervals lasting ≥6 weeks were not uncommon, with 8% of intervals lasting ≥18 weeks; while this may partly reflect limitations of the dataset, it may also reflect unique treatment plans tailored for specific situations and should be more closely evaluated in future studies.

### 4.3 Longitudinal clinical efficacy and durability

The clinical outcomes observed in our cohort were broadly consistent with the efgartigimod clinical trial programs (10, 11, 24, 25), while also providing unique insights into the durability of response in practice. The rapid onset of benefit and the magnitude of the maximum MG-ADL reduction mirrored the efficacy seen in the initial cycles of the pivotal ADAPT trials.

Notably, the trend of sustained disease control observed in our study, reflected by the high proportion of follow-up time spent in CMI, differs from the intentionally cyclical response reported in the pivotal ADAPT trial (10). Instead, this durability is highly consistent with findings from the open-label ADAPT-SC+ study (11), where individualized dosing was similarly employed to maintain long-term clinical benefit. Overinterpretation should be cautioned, as the analytic cohort in this study was enriched with patients starting efgartigimod in 2023–2024, with fewer patients starting in 2025, suggesting enrichment for patients with longer observation time. Nevertheless, nearly 80% of patients spent more than 75% of their follow-up time maintaining CMI, suggesting substantial durability.

A critical finding of our study was the consistency of this durable response across most evaluated subgroups (i.e., age, sex, prior gMG treatment, efgartigimod treatment interval and duration). The only exception was baseline disease severity. Patients with more severe disease demonstrated a more durable CMI response compared to those with milder disease. This difference is well-explained by a measurement floor effect in the mild cohort (mean baseline MG-ADL ≈ 2.7), for whom the required 2-point improvement for CMI necessitated achieving a state consistent with MSE, making their CMI status highly sensitive to minor fluctuations. Despite this sensitivity, the clinical benefit for the mild-disease group remained robust, with approximately 70% of follow-up time maintained in CMI.

These findings collectively address a key question for efgartigimod therapy in gMG, which is whether the individualized nature of dosing impacts the durability of clinical benefit. Our data indicate that despite variability in dosing patterns, consistent and meaningful long-term benefit can be expected with efgartigimod. It should be noted that subgroup analyses were unadjusted. Using alternative datasets with additional information, drivers of better outcomes with efgartigimod should continue to be evaluated, particularly around the timing of initiation (sooner after diagnosis, before exposure to immunosuppressants, etc.) and concomitant treatment strategies.

### 4.4 Deep response and clinical goals, and the impact of baseline severity

MSE is a key treatment goal in gMG and has become increasingly achievable for more patients with targeted therapies (26). Among the large and heterogeneous cohort in this study, 47% achieved MSE at least once, which is largely consistent with that reported in the ADAPT/ADAPT+ trials (10, 25).

Our subgroup analyses (unadjusted) suggested that baseline disease severity was a critical determinant of how clinical benefit manifested. While patients with more severe baseline symptoms demonstrated the greatest magnitude of MG-ADL improvement, those with milder baseline disease (MG-ADL ≤ 4) were most likely to achieve MSE (75.1%). It is expected that patients with lower baseline scores have greater potential to reach MSE. Nevertheless, this high rate of achievement is clinically relevant, as few of these patients (<20%) were in an MSE state prior to treatment. The inclusion of this mild-disease cohort is an important feature of this study, as the rationale for efgartigimod initiation in clinical practice may be multifaceted, including tolerability considerations and patient preferences.

This high rate of MSE in a mild-disease population challenges the common view that targeted therapies should be reserved only for severe or refractory disease. This finding is compelling as it converges with emerging evidence from separate studies examining efgartigimod outcomes among patients with new-onset gMG, where similarly high MSE rates have been reported (27–30). The alignment of these findings from 2 different “early-stage” patient populations (one defined by mild severity, the other by recent onset) collectively suggests that earlier placement of efgartigimod in gMG treatment paradigms may provide more patients an opportunity to achieve this ambitious clinical goal.

Importantly, our analyses on the best observed response should be interpreted in the context of this study’s design based on unstructured clinical practice data. While these best-state metrics do not represent a sustained outcome for all patients, they served a critical methodological purpose in this large and complex dataset. They provided a consistent framework to evaluate the maximal potential for improvement in all 3,326 patients, overcoming the challenge of inconsistent assessment schedules that are incongruous for traditional fixed-timepoint analyses. While these results should not be interpreted alone, when paired with other longitudinal results, they support a larger picture of efgartigimod’s potential to induce a deep and well-maintained response for the majority of patients on active therapy.

### 4.5 Strengths and limitations

A unique strength of this study is its large and diverse cohort, which enhances the generalizability of our findings. Beyond the broad spectrum of clinical severity and treatment experience, the PSP cohort represents a wide cross-section of the US healthcare landscape, sourced from over 1800 prescribers across all 50 US states and including patients with diverse insurance coverage. This robust representation allows for a more reliable interpretation of treatment patterns than do smaller, single-center reports.

However, several important limitations must be acknowledged. The PSP-based dataset lacks clinical details like duration of gMG, detailed treatment history, and changes in concomitant therapies, precluding the ability to evaluate the potential to taper or eliminate other gMG treatments. Because of this limitation, overinterpretation of subgroup analyses based on the unadjusted descriptive statistics should be avoided. Second, the voluntary nature of the PSP limited the ability to accurately assess treatment discontinuations, as patients who stop therapy may become passively lost to follow-up. Third, our analysis identified a strong association between longer treatment duration and favorable outcomes, which requires careful interpretation. This finding is expected and highly likely to be influenced by survivorship bias. Because patients experiencing durable responses may be inherently more likely to persist with therapy, the overall analytic cohort is likely enriched with favorable responders. However, a sensitivity analysis of a static sub-cohort treated for ≥18 months showed deepening clinical improvement over time. This indicates that while survivorship bias exists, longer treatment duration may be independently associated with favorable clinical benefit. Nevertheless, as a descriptive analysis of real-world data, this study cannot establish direct causal relationships. Finally, the inclusion criteria for this study (requiring patients to have a minimum of 4 post-efgartigimod MG-ADL assessments captured in the PSP) also introduced selection bias toward patients with longer treatment experience, supported by the higher number of efgartigimod cycles completed by the analytic cohort compared to patients who were excluded. Nevertheless, this study provides a detailed and valuable description of dosing patterns and clinical outcomes among a large, diverse cohort of persistent efgartigimod users in the US, based on secondary analysis using available data which circumvented additional burden on patients.

## 5 Conclusion

This analysis suggests that the efficacy of efgartigimod observed in clinical trials may translate to meaningful benefit for a broad US patient population. The rapid, deep, and sustained responses observed support efgartigimod’s potential role within the treatment landscape for gMG. The durability of benefit across diverse patient subgroups further highlights its applicability across heterogenous disease presentations. Taken together, these findings indicate that efgartigimod is associated with favorable outcomes in routine clinical practice, with the convergence of proactive strategy, stability, and depth of response contributing to positive patient outcomes.

## Data Availability

The original contributions presented in the study are included in the article/Supplementary material, further inquiries can be directed to the corresponding author.
