# Supplementary material for: Assessing efgartigimod dosing patterns and Myasthenia Gravis Activities of Daily Living outcomes in clinical practice: results from a large patient support program database
Source: Front Neurol. 2026 Jul 30;17:1881758. doi: 10.3389/fneur.2026.1881758 (PMC13470747; doi:10.3389/fneur.2026.1881758)
Supplement: Supplementary file 1 [file Supplementary_file_1.pdf]

Supplementary Material

Supplementary Table 1. Baseline characteristics of analytic cohort and excluded cohort

	**Excluded**	**Study cohort**	***p*[a]**
**Cohort size, n**	5,595	3,326	-
**Age, years[b]**			
Mean (SD)	65.5 (15.7)	65.7 (14.9)	0.311
Median (IQR) (range)	69 (56-77) (18-89)	69 (58-77) (18-89)	
**Sex, n (%)**			
Male	2,859 (51.1)	1,744 (52.4)	0.408
Female	2,268 (40.5)	1,323 (39.8)	
Unknown	468 (8.4)	259 (7.8)	
**Prescriber US region, n (%)**			
South	2,402 (42.9)	1,477 (44.4)	<0.001
Midwest	1,319 (23.6)	538 (16.2)	
West	866 (15.5)	646 (19.4)	
Northeast	853 (15.2)	529 (15.9)	
Not available	155 (2.8)	136 (4.1)	
**Insurance, n (%)[c]**			
Commercial	1,874 (33.5)	1,042 (31.3)	0.009
Government	3,839 (68.6)	2,393 (71.9)	
Other	162 (2.9)	122 (3.7)	
**Prior gMG treatments, n (%)[d]**			
AChEi	2,654 (47.4)	1,454 (43.7)	0.261
Any NSIST	1,106 (19.8)	680 (20.4)	
Any GC	3,152 (56.3)	1,765 (53.1)	
Any advanced therapy[e]	2,943 (52.6)	1,668 (50.2)	
**Baseline MG-ADL score[f]**			
Mean (SD)	8.1 (3.7)	8.2 (3.8)	0.24
Median (IQR) (range)	8 (5-11) (0-23)	8 (5.3-11.0) (0-22)	
Distribution, n (%)			
0-4 points	539 (17.6)	563 (16.9)	0.626
5-9 points	1,476 (48.3)	1,584 (47.6)	
10-15 points	955 (31.2)	1,088 (32.7)	
16-24 points	87 (2.8)	91 (2.7)	
**Time of efgartigimod initiation, n (%)**			
2022	1,452 (26.0)	686 (20.6)	<0.001
2023	1,052 (18.8)	1,228 (36.9)	
2024	1,004 (17.9)	917 (27.6)	
2025	2,087 (37.3)	495 (14.9)	

[a]*p*-values were calculated using independent t-test for continuous variables and Chi-square test for categorical variables. *p*-values of <0.05 were considered statistically significant.
[b]Age information was available for only 5594/5595 and all 3326/3326 patients.
[c]Categories may not add up to the total as patients may have been tagged to multiple payers.
[d]Prior treatment information was available for only 4640/5595 and 2700/3326 patients and was self-reported. Proportions may not add up to 100% as patients could have had multiple concomitant prior therapies. Additionally, some patients may not have listed all their prior treatments, e.g., only their most recent.
[e]Advanced therapies included IVIG/SCIG, PLEX, C5 inhibitors, FcRn blockers, and rituximab.
[f]Baseline scores were available for only 3057/5595 patients and all 3326/3326 patients. The most recent score captured on or before EFG initiation is considered as the baseline score
AChEi, acetylcholinesterase inhibitor; FcRn, neonatal Fc receptor; GC, glucocorticoid; gMG, generalized myasthenia gravis; IQR, interquartile range; IVIG, intravenous immunoglobulin; MG-ADL, Myasthenia Gravis Activities of Daily Living; NSIST, nonsteroidal immunosuppressive therapy; PLEX, plasma exchange; SCIG, subcutaneous immunoglobulin; SD, standard deviation; US, United States.

Supplementary Table 2. Cohort composition over time

	Overall cohort	Subgroups with MG-ADL score available in Month X after EFG initiation			Subgroups with MG-ADL score available in Cycle X after EFG initiation			
		Month 6	Month 12	Month 18	Cycle 3	Cycle 6	Cycle 9	Cycle 12
**N**	**3,326**	**1,454**	**932**	**510**	**2,304**	**1,188**	**636**	**339**
**Age**								
Mean, years (SD)	65.7 (14.9)	65.8 (14.5)	65.9 (14.5)	65.6 (14.2)	65.5 (14.9)	65.6 (14.8)	66.1 (14.5)	66.2 (14.0)
**Sex**								
Female, n (%)	1,323 (39.8)	576 (39.6)	362 (38.8)	188 (36.9)	913 (39.6)	470 (39.6)	241 (37.9)	115 (33.9)
**Prior gMG treatments, n (%)[b]**								
AChEi	1,454 (43.7)	606 (41.7)	348 (37.3)	174 (34.1)	1,005 (43.6)	460 (38.7)	221 (34.7)	116 (34.2)
Any NSIST	680 (20.4)	291 (20.0)	194 (20.8)	131 (25.7)	469 (20.4)	282 (23.7)	169 (26.6)	108 (31.9)
Any GC	1,765 (53.1)	760 (52.3)	500 (53.6)	257 (50.4)	1,213 (52.6)	614 (51.7)	339 (53.3)	199 (58.7)
Any advanced therapy[c]	1,668 (50.2)	724 (49.8)	463 (49.7)	268 (52.5)	1,154 (50.1)	620 (52.2)	342 (53.8)	202 (59.6)
**Efgartigimod formulation used at any time, n (%)[d]**								
IV only	2,106 (63.3)	913 (62.8)	597 (64.1)	342 (67.1)	1,439 (62.5)	722 (60.8)	393 (61.8)	204 (60.2)
SC only	559 (16.8)	232 (16.0)	99 (10.6)	16 (3.1)	370 (16.1)	114 (9.6)	16 (2.5)	4 (1.2)
Both	661 (19.9)	309 (21.3)	236 (25.3)	152 (29.8)	495 (21.5)	352 (29.6)	227 (35.7)	131 (38.6)
**Baseline MG-ADL score**								
Mean (SD)	8.2 (3.8)	8.2 (3.6)	8.2 (3.6)	8.3 (3.8)	8.2 (3.7)	8.3 (3.7)	8.4 (3.7)	8.7 (3.9)
Median (IQR) (range)	8 (5.3-11.0) (0-22)	8 (6-11) (0-21)	8 (6-10) (0-18)	8 (6-11) (0-20)	8 (6-11) (0-21)	8 (6-11) (0-21)	8 (6-11) (0-21)	8 (6-11) (0-21)
**Time of EFG initiation, n (%)**								
2022	686 (20.6)	270 (18.6)	260 (27.9)	210 (41.2)	459 (19.9)	353 (29.7)	266 (41.8)	190 (56.0)
2023	1,228 (36.9)	610 (42.0)	390 (41.8)	227 (44.5)	874 (37.9)	487 (41.0)	283 (44.5)	140 (41.3)
2024	917 (27.6)	386 (26.5)	276 (29.6)	73 (14.3)	673 (29.2)	322 (27.1)	87 (13.7)	9 (2.7)
2025	495 (14.9)	188 (12.9)	6 (0.6)	0 (0)	298 (12.9)	26 (2.2)	0 (0)	0 (0)
**Best MG-ADL score in Cycle 1**								
Mean (SD)	4.3 (3.3)	4.2 (3.3)	4.1 (3.1)	4.1 (3.3)	4.4 (3.3)	4.4 (3.3)	4.2 (3.1)	4.5 (3.3)
**CMI**								
Patients achieving CMI anytime during follow-up, n (%)	3,006 (90.4)	1,349 (92.8)	874 (93.8)	475 (93.1)	2,117 (91.9)	1,111 (93.5)	604 (95.0)	323 (95.3)
**Cycle intervals**								
Cycle interval length, in weeks Median (IQR) (range)	5 (4.0-9.1) (0-126.6)	5 (4.0-9.0) (0-107.9)	5.1 (4.0-9.1) (0-104.3)	5.1 (4.0-9.6) (0-104.3)	5.1 (4.0-9.3) (0-126.6)	5 (4.0-9.0) (0-107.9)	5 (4.0-8.1) (0-105.3)	5 (4.0-7.3) (0-61)

[a]Categories may not add up to the total as patients may be tagged to multiple payers.

[b]Prior treatments information was only available for 2700/3326 patients and were self-reported. Proportions may not add up to 100% as patients could have had multiple concomitant prior therapies. Additionally, some patients may not have listed all their prior treatments, e.g., only their most recent.

[c]Advanced therapies included IVIG/SCIG, PLEX, C5 inhibitors, FcRn blockers, and rituximab.

[d]Both efgartigimod SC and pre-filled syringes (PFS) were considered under the SC formulation category.

AChEi, acetylcholinesterase inhibitor; FcRn, neonatal Fc receptor; GC, glucocorticoid; gMG, generalized myasthenia gravis; IQR, interquartile range; IV, intravenous' IVIG, intravenous immunoglobulin; MG-ADL, Myasthenia Gravis Activities of Daily Living; NSIST, nonsteroidal immunosuppressive therapy; PLEX, plasma exchange; SC, subcutaneous; SCIG, subcutaneous immunoglobulin; SD, standard deviation.

Supplementary Figure 1. MG-ADL response by month and by cycle (all available scores)

(A) Mean MG-ADL response from baseline, by month since efgartigimod initiation

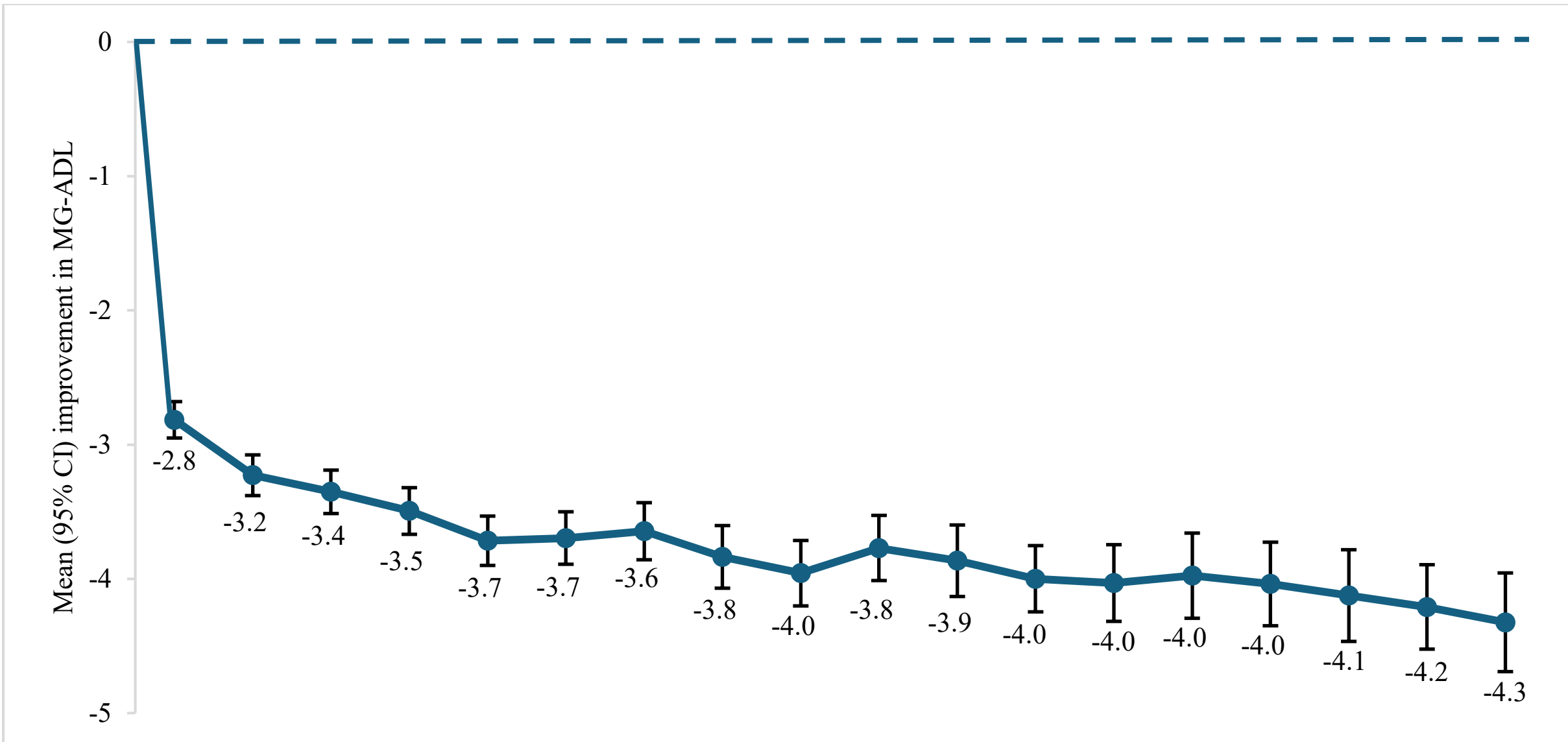

**Month**	1	2	3	4	5	6	7	8	9	10	11	12	13	14	15	16	17	18
**n**	2264	2058	1916	1694	1590	1454	1320	1181	1078	1006	876	932	777	645	669	592	576	510

(B) Mean MG-ADL response from baseline, by efgartigimod treatment cycle

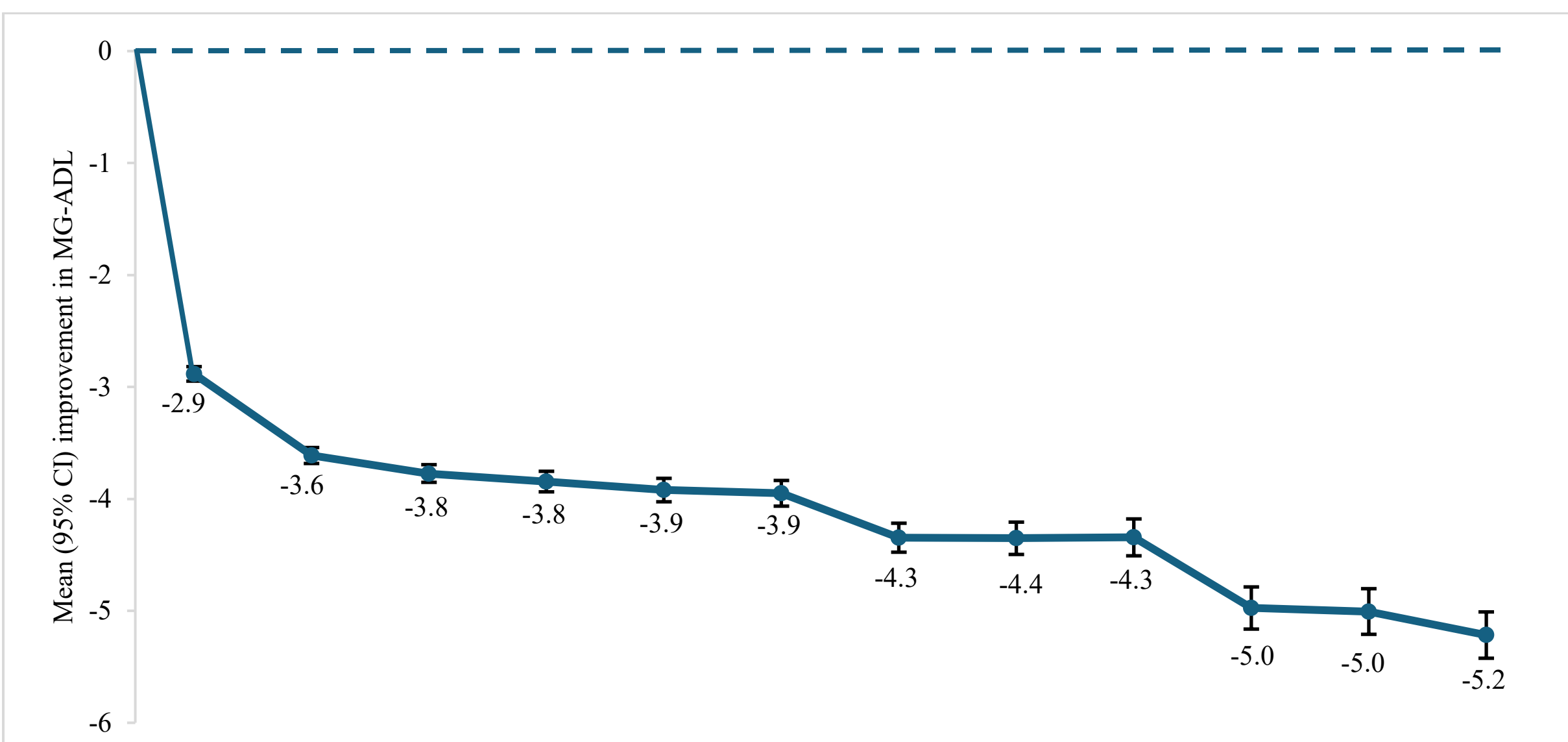

**Cycle**	1	2	3	4	5	6	7	8	9	10	11	12
**n**	2902	2676	2304	1823	1467	1188	956	787	636	522	401	339

MG-ADL, Myasthenia Gravis Activities of Daily Living.

Supplementary Figure 2. MG-ADL response by month and by cycle (among sub-cohort with at least 18 months of continuous efgartigimod exposure; n=1455)

(A) Mean best MG-ADL response from baseline, by month since efgartigimod initiation

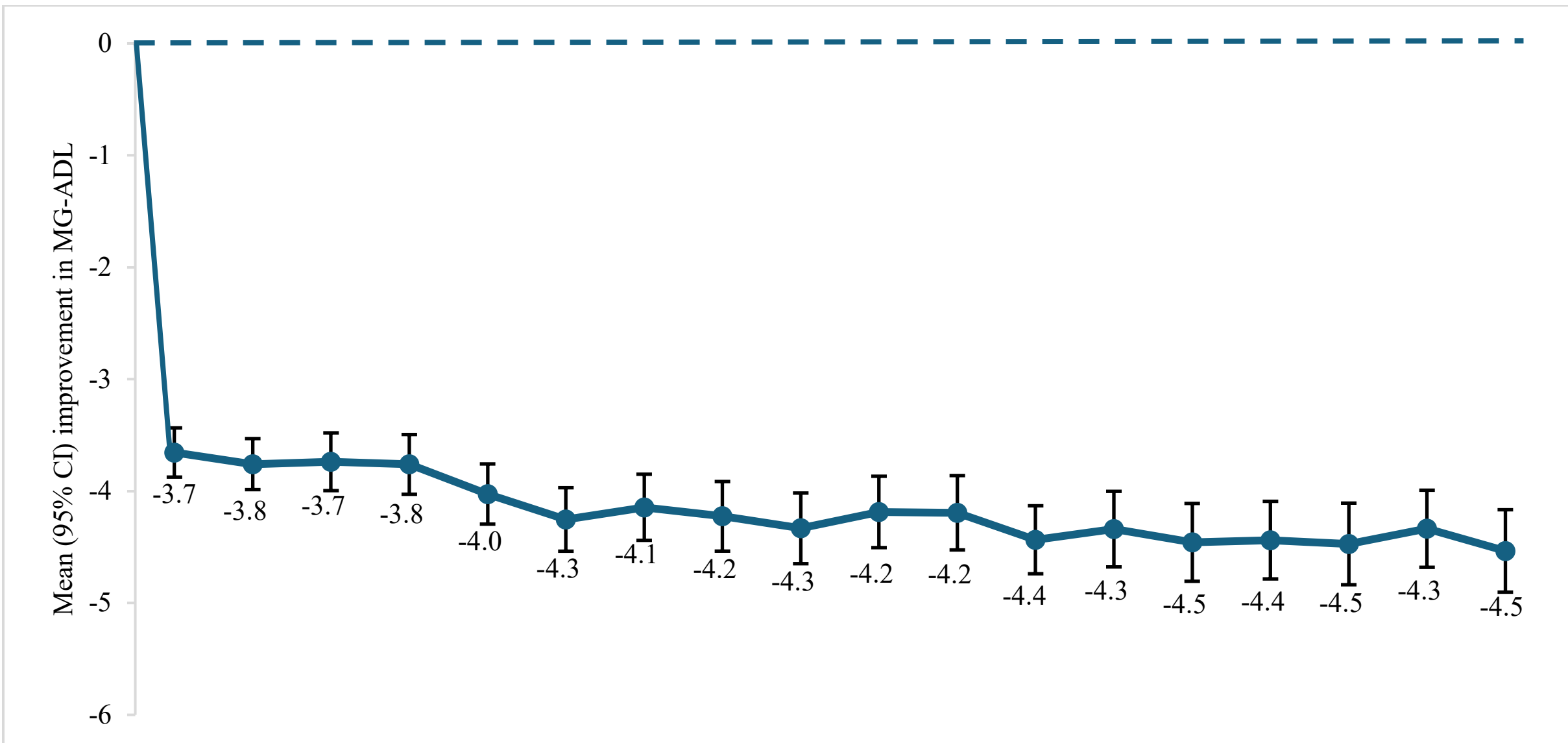

**Month**	1	2	3	4	5	6	7	8	9	10	11	12	13	14	15	16	17	18
**n**	837	780	725	688	681	658	642	616	597	563	552	602	523	467	502	489	496	458

(B) Mean best MG-ADL response from baseline, by efgartigimod treatment cycle

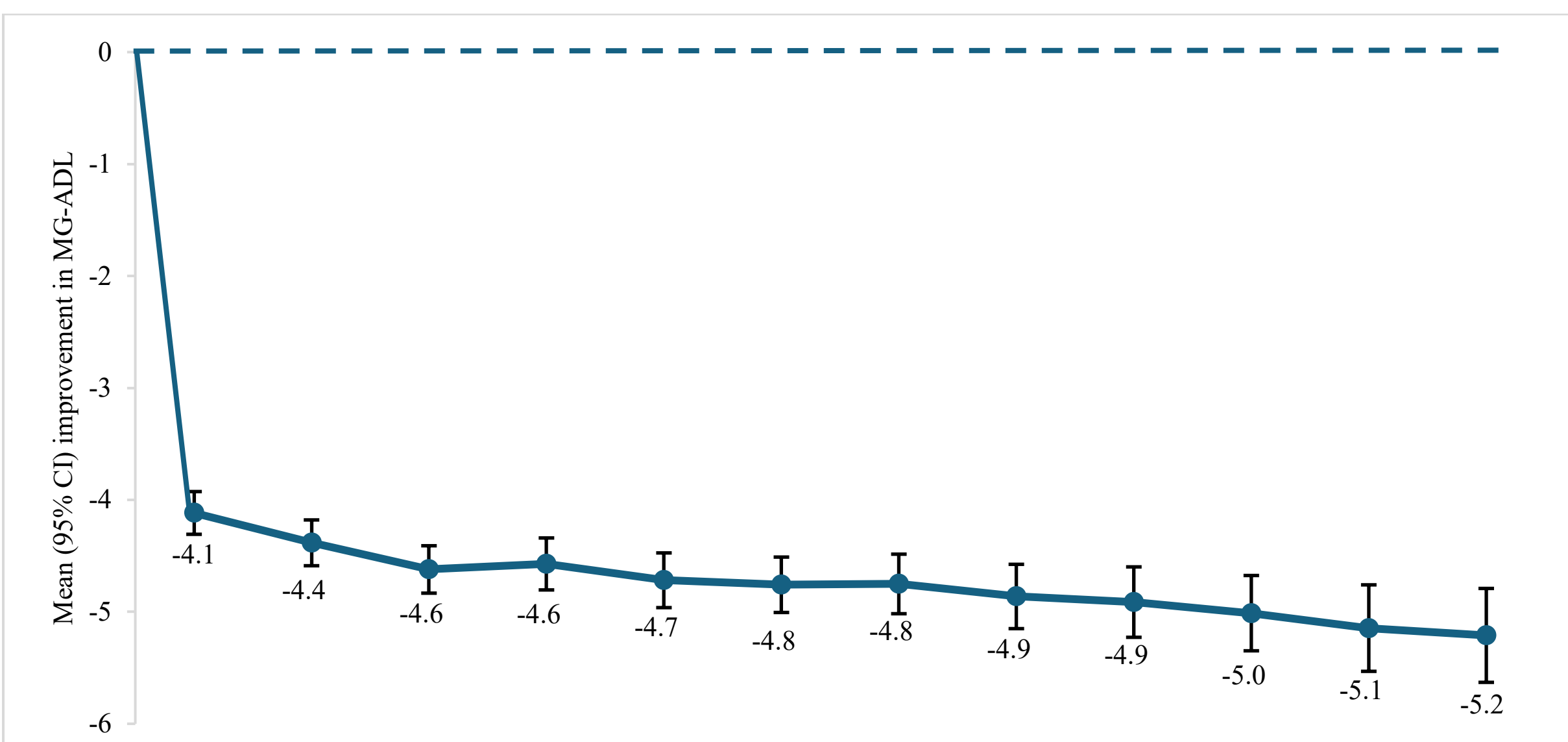

**Cycle**	1	2	3	4	5	6	7	8	9	10	11	12
**n**	1154	1126	1099	1015	940	861	779	693	597	506	399	337

MG-ADL, Myasthenia Gravis Activities of Daily Living

Supplementary Table 3. Durability of clinical response (additional data)

Patients who achieved CMI at least once, with MG-ADL available after achieving CMI, n (%)[a]		2,964 (98.6)
**Cohort-level statistics**		
Time between MG-ADL assessments after first CMI, days	Mean (SD)	55.6 (62.2)
	Median (IQR)	37 (19.5-69.0)
Number of MG-ADL assessments available after first CMI, per patient	Mean (SD)	8.3 (7.8)
	Median (IQR)	6 (4-10)
Number of MG-ADL assessments that were CMI after first CMI, per patient	Mean (SD)	6.8 (7.0)
	Median (IQR)	5 (3-9)
**Patient-level distribution, n (%)**		
Proportion of patients by time from efgartigimod initiation to first CMI		
Less than 3 months		2,337 (78.8)
3-<6 months		373 (12.6)
6+ months		254 (8.6)
Proportion of patients with x% of their MG-ADL assessments after first CMI maintaining CMI		
100%		1,560 (52.6)
≥90%		1,721 (58.1)
≥75%		2,172 (73.3)

[a]2964 patients out of the analytic cohort of N=3326 were eligible for this analysis. Of the analytic cohort, 3006 (90.4%) patients achieved CMI at least once, and 2964 (89.1%) had at least 1 MG-ADL score recorded after their first CMI achievement. 42 patients were excluded as they did not have any additional MG-ADL scores recorded after their first CMI.

CMI, clinically meaningful improvement; IQR, interquartile range; MG-ADL, Myasthenia Gravis Activities of Daily Living; SD, standard deviation.

Supplementary Table 4. MG-ADL subdomains

Subdomain (range)	Mean baseline score	Largest observed response	
		Mean score improvement from baseline	Percent improvement from baseline
Ocular (0-6)	2.66	1.95	73.2%
Bulbar (0-9)	2.30	1.90	82.6%
Limb/gross motor (0-6)	2.32	1.48	63.5%
Respiratory (0-3)	0.89	0.55	62.2%

MG-ADL, Myasthenia Gravis Activities of Daily Living.

Supplementary Table 5: MG-ADL response by subgroups: pairwise *p*-values

By age

Mean magnitude of largest observed MG-ADL from baseline, by age

Age, years	18-<50	50-<65	≥65
18-<50	-	0.834	<0.001
50-<65	0.834	-	<0.001

Proportion achieving MSE, by age

Age, years	18-<50	50-<65	≥65
18-<50	-	0.200	0.222
50-<65	0.200	-	0.770

Mean proportion of time spent in CMI, by age

Age, years	18-<50	50-<65	≥65
18-<50	-	0.276	0.201
50-<65	0.276	-	0.972

By sex

Mean magnitude of largest observed MG-ADL from baseline, by sex

Sex	Male	Female	Unknown
Male	-	0.001	0.602
Female	0.001	-	0.181

Proportion achieving MSE, by sex

Sex	Male	Female	Unknown
Male	-	<0.001	0.847
Female	<0.001	-	0.057

Mean proportion of time spent in CMI, by sex

Sex	Male	Female	Unknown
Male	-	0.699	0.094
Female	0.699	-	0.147

By prior gMG treatment

Mean magnitude of largest observed MG-ADL from baseline, by prior gMG treatment

Prior gMG treatment[a]	Any advanced therapy	Any other gMG treatments captured	Not known/ thymectomy
Any advanced therapy	-	0.185	0.739
Any other gMG treatments captured	0.185	-	0.156

Proportion achieving MSE, by prior gMG treatment

Prior gMG treatment[a]	Any advanced therapy	Any other gMG treatments captured	Not known/ thymectomy
Any advanced therapy	-	0.105	0.934
Any other gMG treatments captured	0.105	-	0.156

Mean proportion of time spent in CMI, by prior gMG treatment

Prior gMG treatment[a]	Any advanced therapy	Any other gMG treatments captured	Not known/ thymectomy
Any advanced therapy	-	0.901	0.961
Any other gMG treatments captured	0.901	-	0.880

By baseline MG-ADL score

Mean magnitude of largest observed MG-ADL from baseline, by baseline MG-ADL score

Baseline MG-ADL score	0-4	5-9	10-24
0-4	-	<0.001	<0.001
5-9	<0.001	-	<0.001

Proportion achieving MSE, by baseline MG-ADL score

Baseline MG-ADL score	0-4	5-9	10-24
0-4	-	<0.001	<0.001
5-9	<0.001	-	<0.001

Mean proportion of time spent in CMI, by baseline MG-ADL score

Baseline MG-ADL score	0-4	5-9	10-24
0-4	-	<0.001	<0.001
5-9	<0.001	-	<0.001

By average interval length

Mean magnitude of largest observed MG-ADL from baseline, by average gap length

Average gap length[b]	<4 weeks	4-6 weeks	≥6 weeks
<4 weeks	-	0.862	0.719
4-6 weeks	0.862	-	0.231

Proportion achieving MSE, by average gap length

Average gap length[b]	<4 weeks	4-6 weeks	≥6 weeks
<4 weeks	-	0.123	0.793
4-6 weeks	0.123	-	0.003

Mean proportion of time spent in CMI, by average gap length

Average gap length[b]	<4 weeks	4-6 weeks	≥6 weeks
<4 weeks	-	0.687	0.526
4-6 weeks	0.687	-	0.636

By number of cycles completed

Mean magnitude of largest observed MG-ADL from baseline, by number of efgartigimod cycles completed

Number of efgartigimod cycles completed	1	2	3	4	≥5
1	-	0.114	0.041	0.087	<0.001
2	0.114	-	0.574	0.850	<0.001
3	0.041	0.574	-	0.717	<0.001
4	0.087	0.850	0.717	-	<0.001

Proportion achieving MSE, by number of efgartigimod cycles completed

Number of efgartigimod cycles completed	1	2	3	4	≥5
1	-	0.604	0.284	0.231	<0.001
2	0.604	-	0.077	0.060	<0.001
3	0.284	0.077	-	0.934	0.003
4	0.231	0.060	0.934	-	0.008

Mean proportion of time spent in CMI, by number of efgartigimod cycles completed

Number of efgartigimod cycles completed	1	2	3	4	≥5
1	-	0.283	0.662	0.632	0.707
2	0.283	-	0.485	0.087	0.274
3	0.662	0.485	-	0.313	0.853
4	0.632	0.087	0.313	-	0.263

By treatment duration

Mean magnitude of largest observed MG-ADL from baseline, by efgartigimod treatment duration

Efgartigimod treatment duration[c]	<3 months	3-<6 months	6-<12 months	12-<18 months	≥18 months
<3 months	-	0.318	0.033	0.006	<0.001
3-<6 months	0.318	-	0.067	0.005	<0.001
6-<12 months	0.033	0.067	-	0.161	<0.001
12-<18 months	0.006	0.005	0.161	-	<0.001

Proportion achieving MSE, by efgartigimod treatment duration

Efgartigimod treatment duration[c]	<3 months	3-<6 months	6-<12 months	12-<18 months	≥18 months
<3 months	-	0.249	0.003	0.001	<0.001
3-<6 months	0.249	-	0.002	0.001	<0.001
6-<12 months	0.003	0.002	-	0.552	<0.001
12-<18 months	0.001	0.002	0.552	-	<0.001

Mean proportion of time spent in CMI, by efgartigimod treatment duration

Efgartigimod treatment duration[c]	<3 months	3-<6 months	6-<12 months	12-<18 months	≥18 months
<3 months	-	0.853	0.534	0.759	0.763
3-<6 months	0.853	-	0.111	0.344	0.306
6-<12 months	0.534	0.111	-	0.423	0.292
12-<18 months	0.759	0.344	0.423	-	0.967

p-values were calculated using independent t-test for continuous variables, and Chi-square test for categorical variables. *p*-values of <0.05 were considered statistically significant.
Multiplicity adjustments were not applied, as subgroup analyses were exploratory in nature (descriptive associations).
[a]Prior gMG treatment information was only available for 2700/3326 patients and was self-reported. Advanced therapies included IVIG/SCIG, PLEX, C5 inhibitors, FcRn blockers, and rituximab. Patients who reported any prior treatments that were not advanced therapies (any AChEi, any steroids, and/or any NSISTs) were grouped into "Any other gMG treatments captured" for this analysis.
[b]Average of all cycle gaps at patient level.
[c]Difference between efgartigimod initiation date and the latest efgartigimod infusion date or MG-ADL capture date, whichever was recorded the most recently.
AChEi, acetylcholinesterase inhibitor; CMI, clinically meaningful improvement; FcRn, neonatal Fc receptor; gMG, generalized myasthenia gravis; IVIG/SCIG, intravenous/subcutaneous immunoglobulin; MG-ADL, Myasthenia Gravis Activities of Daily Living; MSE, minimal symptom expression; NSIST, nonsteroidal immunosuppressive therapy; PLEX, plasma exchange.
